# 4*th* Generation Biomaterials Based on PVDF-Hydroxyapatite Composites Produced by Electrospinning: Processing and Characterization

**DOI:** 10.3390/polym14194190

**Published:** 2022-10-06

**Authors:** Gabriel Grube dos Santos, Milena Schroeder Malherbi, Natália Silva de Souza, Gabriel Batista César, Tania Toyomi Tominaga, Ricardo Yoshimitsu Miyahara, Patrícia de Souza Bonfim de Mendonça, Daniela Renata Faria, Jaciele Márcia Rosso, Valdirlei Fernandes Freitas, Wilson Ricardo Weinand, Gustavo Sanguino Dias, Ivair Aparecido Santos, Luiz Fernando Cotica, Taiana Gabriela Moretti Bonadio

**Affiliations:** 1 Graduate Program in Applied Chemistry, Midwestern Paraná State University, Guarapuava 85040167, PR, Brazil; 2 Department of Physics, Midwestern Paraná State University, Guarapuava 85040167, PR, Brazil; 3 Department of Chemistry, Midwestern Paraná State University, Guarapuava 85040167, PR, Brazil; 4 Department of Clinical Analysis and Biomedicine, State University of Maringá, Maringá 87020900, PR, Brazil; 5 Graduate Program in Biosciences and Pathophysiology, State University of Maringá, Maringá 87020900, PR, Brazil; 6 Department of Physics, State University of Maringá, Maringá 87020900, PR, Brazil

**Keywords:** scaffold, biomaterial, poly(vinylidene fluoride), apatite

## Abstract

Biomaterials that effectively act in biological systems, as in treatment and healing of damaged or lost tissues, must be able to mimic the properties of the body’s natural tissues in its various aspects (chemical, physical, mechanical and surface). These characteristics influence cell adhesion and proliferation and are crucial for the success of the treatment for which a biomaterial will be required. In this context, the electrospinning process has gained prominence in obtaining fibers of micro- and nanometric sizes from polymeric solutions aiming to produce scaffolds for tissue engineering. In this manuscript, poly(vinylidene fluoride) (PVDF) was used as a polymeric matrix for the manufacture of piezoelectric scaffolds, exploring the formation of the β-PVDF piezoelectric phase. Micro- and nanometric hydroxyapatite (HA) particles were incorporated as a dispersed phase in this matrix, aiming to produce multifunctional composite membranes also with bioactive properties. The results show that it is possible to produce membranes containing micro- and nanofibers of the composite by the electrospinning process. The HA particles show good dispersion in the polymer matrix and predominance of β-PVDF phase. Also, the composite showed apatite growth on its surface after 21 days of immersion in simulated body fluid (SBF). Tests performed on human fibroblasts culture revealed that the electrospun membranes have low cytotoxicity attesting that the composite shows great potential to be used in biomedical applications as bone substitutions and wound healing.

## 1. Introduction

The production of materials that are capable of making a partial or complete replacement of components of living matter (biomaterials) has been widely studied in recent decades [1,2,3].

In this context, bioelectrical signals have been associated with the control of different cellular behaviors such as differentiation, proliferation, apoptosis (cell death), migration, among others [4]. Electrical stimulation can also have an effect on the healing and regeneration of tissues such as skin, bones and nerves [5]. Therefore, electroactive biomaterials such as the piezoelectric ones have aroused great interest in tissue engineering because of their ability to provide intrinsic electrical stimulation [6]. In fact, biomaterials that are able to mimic the electrical properties and microenvironments in living organisms are called “4th Generation Biomaterials” [7].

Living bone has an intrinsic piezoelectric effect that manages the bone remodeling process [8]. Therefore, the piezoelectric effect should be a relevant parameter to be considered in the development of innovative biomaterials to replace bone tissue. A material that has great potential for such application is the composite that combines the biocompatible piezoelectric polymer poly(vinylidene fluoride) (PVDF) with the bioactive hydroxyapatite (HA) ceramic.

PVDF is a semicrystalline polymer from the fluoropolymer family that present piezoelectricity [9], elevated thermal stability and mechanical strength [10]. PVDF has a simple polymeric structure, with a repeating unit of chemical formula ${{(C}_{2}H_{2}F_{2})}_{n}$, which allows an excellent mechanical response [11]. PVDF has five distinct crystalline phases, namely α, β, γ, δ, and ε. The α phase is the only non-polar phase and is the most abundant among all. The β phase is the one that has the highest electric dipole moment among all the polar phases and is of most interest for applications that exploit the piezoelectricity of PVDF [12,13,14]. The applicability of PVDF is very broad and consistent, ranging from applications in batteries, electromechanical filters, actuators and ultrasonic transducers in biomedical areas [11,15].

Hydroxyapatite (HA), on the other side, is a calcium phosphate with chemical formula ${Ca}_{10}{\left({PO}_{4}\right)}_{6}{\left(OH\right)}_{2}$ that is highly explored in the field of bioceramics, and one of the most used when it comes to biomedical and clinical applications for having great similarity with the crystalline phase of the bone tissue [16]. Hydroxyapatite is an osteoconductive, biocompatible, bioactive and thermodynamically stable material [17], and when combined with biocompatible polymers (PVDF, polylactic acid (PLA), polycaprolactone (PCL), among others) it becomes a material with great potential for development of composite scaffolds [18,19,20].

The electrospinning technique is a very effective technique in the production of scaffolds formed from micro- and nanometric polymeric fibers that could be applied in air filtration and facemasks, wound dressings, optical fiber sensors, water purification, blood vessels, axons, adsorption, photosensitive materials, electronics, drug delivery systems and various other fields of science and technology [21,22,23,24].

The single-fluid blending electrospinning technique consists in the application of a potential difference between a metallic conductor and the needle of a syringe that contains a polymeric solution forming nano- and microfibers. On the other hand, this traditional electrospinning can be differentiated into different sorts, e.g., of double-fluid and three-fluid processes [25]. Based on the spatial positions, there are different types of dual fluid electrospinning, namely coaxial electrospinning and side-by-side electrospinning. The coaxial electrospinning is interesting due to its capability of creating core-shell structures that can provide a desired drug-modified release profiles from more adjustable elements, as well as the properties of polymer matrices. In the study by Lv et al. [26], a kind of hybrid with a coating of crystalline nanoparticles of acyclovir on acyclovir-polyacrylonitrile composites was manufactured using modified coaxial electrospinning processes. Compared to electrospun composite nanofibers from a single fluid mixing process, nanohybrids have shown advantages in modifying acyclovir release profiles in several aspects. A Janus nanofiber dressing with a hierarchical structure has been successfully manufactured using side-by-side electrospinning technology. On the other hand, side-by-side electrospinning technology can be exploited in creating Janus nanofiber dressings with a hierarchical structure, outstanding surface wettability, excellent mechanical properties, and rapid drug release [27]. Three-fluid processes are also possible in electrospinning. In this case, three syringe pumps are used to push the working fluids into the electric field and a tri-axial spinneret for separating the fluids. The types of spinneret are the most important, which directly determine the structures of electrospun nanofibers [28]. Despite the numerous possibilities of electrospinning, the single-fluid blending electrospinning technique is still used due its simplicity. This is the case of the recent study of Liu et al. in which single-fluid blending electrospinning process was combined with the casting film method to fabricate a medicated double-layer hybrid to provide a dual-phase ibuprofen controlled release profile [29].

Biomaterials produced by electrospinning have been used as scaffolds for the growth of cells from different tissues of the body, even hard tissues such as bones [30,31,32]. Furthermore, the electrospinning process is versatile, allowing different materials to be combined in order to create composites capable of meeting different demands. Therefore, in this work, scaffolds of PVDF and HA (PVDF-HA composite) were produced by a simple single-fluid electrospinning method and carefully investigated. In particular, the use of the single-fluid electrospinning technique proved to be advantageous, since it is responsible for inducing the formation of the β-PVDF phase. The obtained scaffolds were analyzed for their morphology, piezoelectric phase (β-phase formation), in vitro bioactivity and cytotoxicity. The scaffolds showed important characteristics and may be potential candidates to act as 4th generation biomaterials to be used in electroactive coatings on bone implants or as smart dressings for the repair of soft tissue in wounds.

## 2. Materials and Methods

### 2.1. Materials

PVDF powder (99.8% purity, M${}_{w}$ = 6.4 × 10${}^{4}$ g/mol) was purchased from Alfa Aesar Company. Hydroxyapatite (Ca${}_{10}$(PO${}_{4}$)${}_{6}$OH${}_{2}$, HA) was obtained from the calcination and milling of bovine bones at 900 ºC, according to the procedure described by Miyahara et al. [33]. The solvent N,N-Dimethylformamide (DMF) was obtained from Dinâmica Company (99.8% purity) and acetone (99.5% purity) was obtained from Anidrol Company.

### 2.2. Scaffolding Processing

Samples processing took place in two stages: The first consisted of optimizing the amounts of PVDF and solvents for good electrospinning. For the second, the proportions defined in the first step were used and different proportions of hydroxyapatite were added. In the first step, the proportions between the solvents of DMF/Acetone were 80/20, 70/30 and 60/40 (*v*/*v*). The proportions of PVDF for these solutions were 18, 16 and 14% (w/*v*), respectively. In the second step, 10, 15 and 20% of HA (HA/PVDF–(*w*/*w*)) were added in relation to the PVDF mass in the samples with 14% PVDF. The sample with 14% of PVDF without HA was used as control group. This proportion of PVDF was the one that presented the best results among the solutions made previously (without HA), regarding the amount of β-PVDF phase and regarding the diameters of the electrospun fibers. Hereafter, these samples, with 0, 10, 15 and 20% of HA will be designated by the abbreviations, P14 (control), P14-H10, P14-H15 and P14-H20, respectively. The mixture was made as follows: HA was incorporated into DMF and kept under stirring for 16 h. Subsequently, this dispersion was heated to 80 ${}^{\circ }$C and the PVDF was slowly added until all the powder was completely dissolved. This mixture was kept under stirring and heating for approximately 4 h. After this period, the heating was turned off and the mixture was allowed to reach room temperature. After that, acetone was added to the mixture, which was stirred for another 30 min. All stirring processes were carried out in closed flasks to avoid evaporation of solvents.

### 2.3. Electrospinning Parameters

The electrospinning solution was placed in the 5 mL syringe equipped with a metal needle (gauge: 22G1 ⅟₄) connected to the positively charged electrode of the high-voltage power supply. The electrospinning device was acquired from the Spin Tech Soluções para Laboratórios company. During the electrospinning process, the voltage was fixed at 16.5 ± 1.5 kV and the tip-to-collector distance at 15 cm. The pumps for delivering the electrospinning solutions were located at an angle of 180${}^{\circ }$ in respect to the rotating collector (270 cm/s) and the delivery rate was 0.83 mL/h (sample without HA) and 0.35 mL/h (sample with HA). The frequency of the rotating collector was 258 RPM. In all cases, the relative humidity was between 45 and 53% and the room temperature between 21 and 25 ${}^{\circ }$C. The electrospinning process was maintained for 2 to 3 h, for all solutions.

### 2.4. Characterizations

Scanning electron microscopy (SEM) was performed with a Hitachi TM3000 XSTREAM2 microscope with accelerating voltage of 15 kV. From the scanning electron microscopy images, measurements of the diameter of 100 fibers of each sample and the pore sizes were estimated by Feret’s diameter (maximum distance between two parallel tangents which reach opposite sides of the object). These analyses were performed using the ImageJ free software [34]. A Tescan VEGA 3 microscope was also used to obtain SEM images with Energy-Dispersive X-ray Spectroscopy (EDS) elemental mapping with accelerating voltage of 15 kV. This microscope is coupled to a detection system by EDS microanalysis, model INCA X-ACT STANDARD, resolution of 129 eV (Oxford). Room temperature structural characterizations were performed by X-ray powder diffraction (XRD) using a SHIMADZU XRD7000-diffractometer (Cu Kα radiation). The Fourier transform infrared spectroscopy (FTIR) characterizations were performed in a spectrophotometer Perkin Elmer Frontier, using an accessory for Attenuated Total Reflectance (ATR) measurements in the range of 4000 and 650 cm${}^{-1}$ (4 cm${}^{-1}$ of resolution). The FTIR also allowed the estimation of the percentage of the β-PVDF phase using the method proposed by Cai et al. [35]. Simulated body fluid (SBF), which simulates the conditions of human body for preliminary bioactivity tests (in vitro tests) [36,37,38,39], was used to investigate the scaffolds’ bioactivity. This technique, firstly developed by Kokubo et al., permits that a bioactive material (Apatite (Ca${}_{5}$(PO${}_{4}$)${}_{3}$(OH,F,Cl))) growth on the samples surface immersed in SBF (by simulating a growing medium similar to that of the human body) [40,41,42].

### 2.5. Cytotoxicity Test

In vitro cytotoxicity tests were conducted on samples P14 (control), P14-H10 and P14-H15 in L929 fibroblast cell. These membranes were chosen because they presented the best results regarding the morphology of the fibers. For the evaluation of direct cytotoxicity, the samples were cut in the form of circles with a diameter of approximately 16.25 mm, equivalent to the size of the 24-well cell culture plates, and ultra violet (UV) sterilized for 1 h (30 min on each side). The samples were then immersed in complete Dulbecco’s Modified Eagle Medium (DMEM) medium (500 μL) supplemented with 10% FBS and 1% Penicillin/Streptomycin in a 24-well polystyrene tissue culture plate and incubated overnight. Subsequently, the culture medium was discarded from the wells and 100 μL of fibroblast cell suspension (2 × 10${}^{5}$ cells/mL) was seeded on top of each sample to allow the cells to adhere to the testing materials. After 3 h of incubation, an additional 400 μL of complete cell-free DMEM medium was added to reach 500 μL of total volume. The negative control consisted of fibroblasts seeded in empty wells without addition of any testing materials while the positive control consisted of seeding the cells in empty wells with 20% dimethyl sulfoxide (DMSO). Samples and controls with fibroblasts were kept at 37 ${}^{\circ }$C in an environment of 5% CO${}_{2}$ and 95% atmospheric air for periods of 24 h, 72 h and 7 days. The culture medium was changed every 3 days. Cell viability was based on the reduction of MTS [3-(4,5-dimethylthiazol-2-yl)-5-(3-carboxymethoxyphenyl)-2-(4-sulfophenyl)-2H-tetrazolium], performed according to the manufacturer. After the incubation times, the culture medium was discarded and the wells were washed 1 × with phosphate buffered saline (PBS). Then, a solution of MTS, prepared in DMEM without phenol red, was added to the wells. After 3 h of incubation in the dark, the optical density was measured at 492 nm on a microplate reader (SpectraMax ^®^ Plus 384). Quantitative results were obtained from the mean and standard deviation for the triplicate wells of each sample. And cell viability was calculated as follows: *% cell viability = AbsA × 100/AbsNC*, where *Abs A* is the metabolic activity of fibroblasts in the presence of test samples P14, P14-H10 or P14-H15, and *AbsNC* is the metabolic activity of fibroblasts in the absence of the compounds, i.e., negative control.

## 3. Results and Discussion

**Figure 1:**
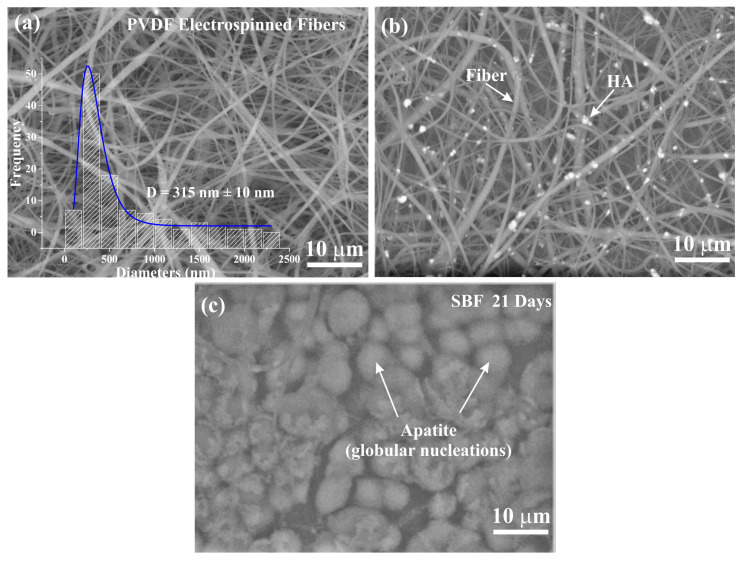
SEM images for (**a**) P14, (**b**) P14-H10 and (**c**) P14-H15 scaffolds (the image was obtained after immersion in SBF 21 days).

Figure 1a shows a SEM image of sample P14 after the electrospinning process. The presence of very continuous fibers without beads is seen. Figure 1a shows a histogram of fiber diameter distribution with an average diameter of 350 nm and narrow dispersion (±10 nm). In Figure 1b a micrograph of P14-H10 sample is shown (backscattered electron detector image was used to highlight the contrast difference between HA and PVDF fibers). It is noted that the incorporation of HA occurred satisfactorily, with good dispersion of these particles in the membranes. For this sample, there was an increase in the average diameter of the fibers in relation to the pure polymeric fiber. The diameter of the fibers varied from 200 to 2200 nm, and the mean and standard deviation obtained through the normal curve were 587 and 365 nm, respectively. For the sample P14-H15 (not shown) the results were very similar. The variation in fiber diameter was from approximately 100 to 2500 nm, with mean and deviation of 577 and 467 nm, respectively. After the electrospinning process, the P14-H20 sample was brittle, making it difficult to remove it from the aluminum foil in which the polymeric solution is collected during the electrospinning process. Deliormnli and Konyali [43] found similar results, where the addition of HA in PCL solution led to an increase in the average diameter of electrospun fibers, compared to pure PCL fibers. Likewise, Tandon et al. [19] observed an increase in the average size of PVDF fibers with incorporation of HA, compared to pure polymer fibers. It was also reported that this may be related to changes in solution properties, caused by the addition of ceramics, such as increased viscosity and possible changes in conductivity and solvent evaporation rate. Thus, it appears that, from the SEM analysis, P14-H10 and P14-H15 samples presented the most satisfactory results. On the other hand, the pore sizes (estimated by the calculation of Feret’s diameter) were similar for all the samples. The P14, P14-H10 and P14-H15 samples present Feret’s diameter of 1.524±1.501μm, 1.279±1.430μm and 1.294±1.202μm, respectively. It must be considered that the SEM image provides two-dimensional data and the calculation of Feret’s diameter does not distinguish fibers that are in different planes in the scaffold. Therefore, the obtained pore sizes may be slightly underestimated. Even so, the values obtained are an estimate of the order of magnitude of pores obtained with electrospun fibers. In fact, MG63 human osteoblasts could be fully adhered and spread on the polycarbonate (PC) surface with 0.2–1 μm pores [44].

Furthermore, fibroblasts cultured on one side of a membrane with a pore size of 1.2μm, were able to contact cells on the other side of the membrane [44]. On the other hand, 3μm pore size scaffold membrane allowed migration of feeder cells, while the 1μm pore size was adequate for growing embryonic stem cells [44]. These data show that the membranes obtained in the present study have good characteristics to serve as a scaffold for several cells.

**Figure 2:**
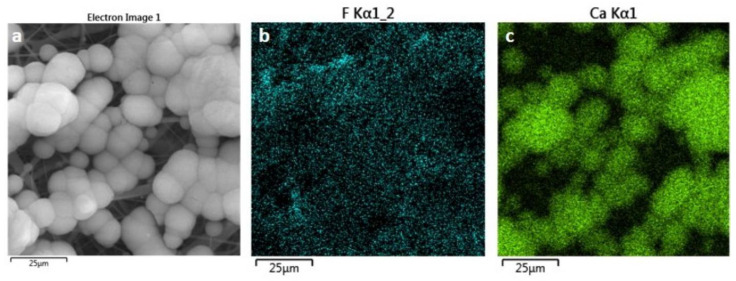
(**a**) SEM images obtained with secondary electrons detector for the P14-H10 sample. (**b**),(**c**) EDS mapping of the region presented in (**a**) for F and Ca elements, respectively.

Figure 1c shows SEM images for the P14-H15 sample after 21 days of immersion in SBF. The image shows the formation of globular structures, characteristic of the apatite growth process in vitro, can be seen [36,37]. On the other hand, we observe some regions without apatite growth in the sample. This demonstrates that, despite the sample showing bioactivity, this did not occur uniformly throughout the sample. Probably, with longer periods of immersion, the sample must be completely covered by this layer of globular nucleations. Confirmation that the nucleated layer on the scaffolds is apatite is shown in the EDS mapping, Figure 2.

**Figure 3:**
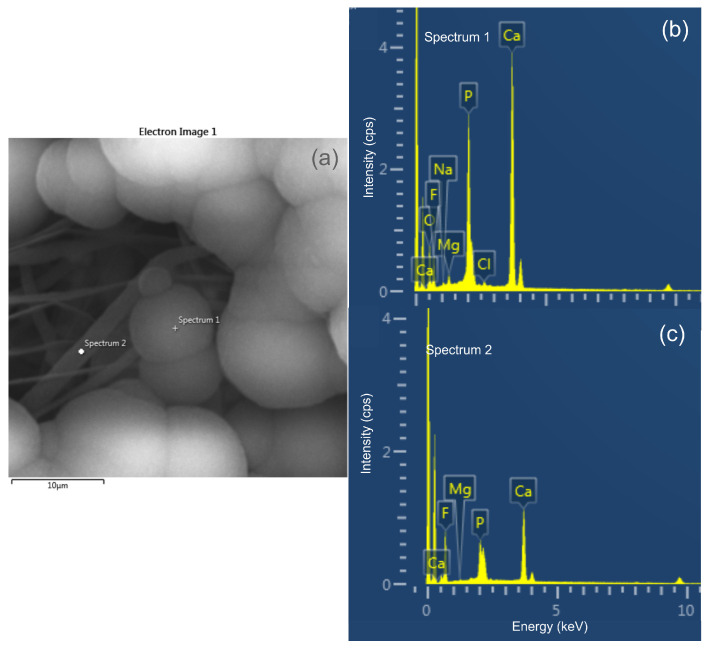
(**a**) SEM image of two different regions: with apatite (Spectrum 1) and without apatite (Spectrum 2) of the P14-H10 scaffold after 21 days of immersion in SBF. (**b**),(**c**) are EDS spectra in the point “Spectrum 1” and “Spectrum 2” of (**a**), respectively.

Figure 2a shows the SEM image (secondary electrons) of the P14-H10 scaffold after 21 days of immersion in SBF. On the electrospun fibers the presence of globular nucleations, characteristic of in vitro apatite growth process is seen. The fluorine element, which is present in the PVDF is observed, in the entire region of the sample, as shown in Figure 2b. On the other hand, the calcium element appears more concentrated in regions where there is nucleation (Figure 3c). The same behavior was observed for the phosphorus element (not shown). The results demonstrate that the nucleated layer on the scaffolds is in fact apatite and not some precipitation of another salt. Figure 3 shows spectra of two different regions of the membrane (with apatite and without apatite) of the P14-H10 scaffold after 21 days of immersion in SBF. In Figure 3a is shown the image of SEM. Figure 3b and c shows the EDS spectra in the point “Spectrum 1” and “Spectrum 2” of the Figure 3a, respectively. One can see that the intensity of the peaks related to calcium (Ca) and phosphorous (P) elements, in comparison to the intensity of the peak of fluorine (F) element, is greater at the region where it is observed the nucleation of apatite. This result unambiguously proves the nucleation of apatite on the scaffold surface.

**Figure 4:**
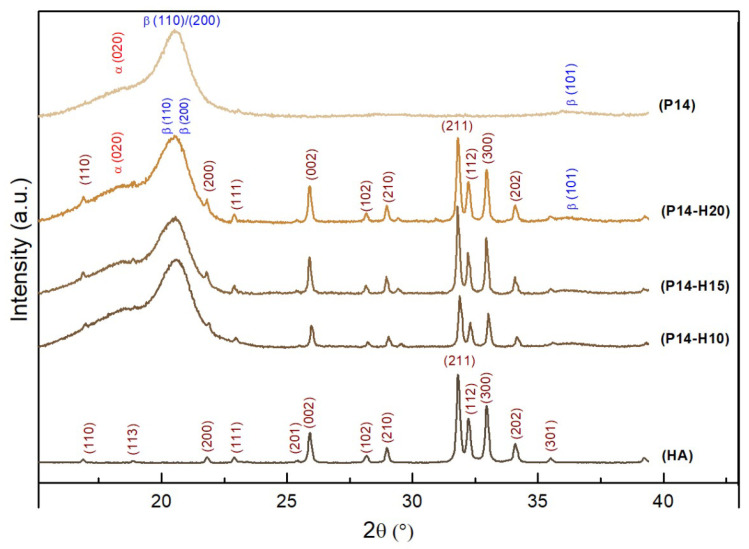
XRD patterns for HA (powder), P14, P14-H10,P14-H15 and P14-H20 (scaffolds) samples.

Figure 4 shows the diffractograms obtained for the HA powder sample and for the P14, P14-H10, P14-H15 and P14-H20 scaffolds. For HA, the diffraction pattern was indexed by Miller of the hexagonal structure of space group *P*63/*m*. PVDF has the chemical formula (-H${}_{2}$C-CF${}_{2}$-)${}_{n}$, where n is the number of monomer repetitions. The α phase was indexed with the *P*21/*c* space group (monoclinic symmetry) and the β phase with the *Cm*2*m* space group (orthorhombic symmetry). In the scaffolds, peaks related to the PVDF phases at 2θ = 18.5${}^{\circ }$ and 20.5${}^{\circ }$ are observed. These peaks correspond to the (020) and (110)/(200) planes and to the α and β phases of the PVDF polymer, respectively [35,45,46]. The diffraction peak that is characteristic of the α-phase appears only as a shoulder for in all three diffractograms, while the β-phase peak appears well-defined. In addition to the polymer peaks, HA diffraction peaks remains evident for three samples. As seen, the formation of new crystalline phases was not observed after electrospinning.

**Figure 5:**
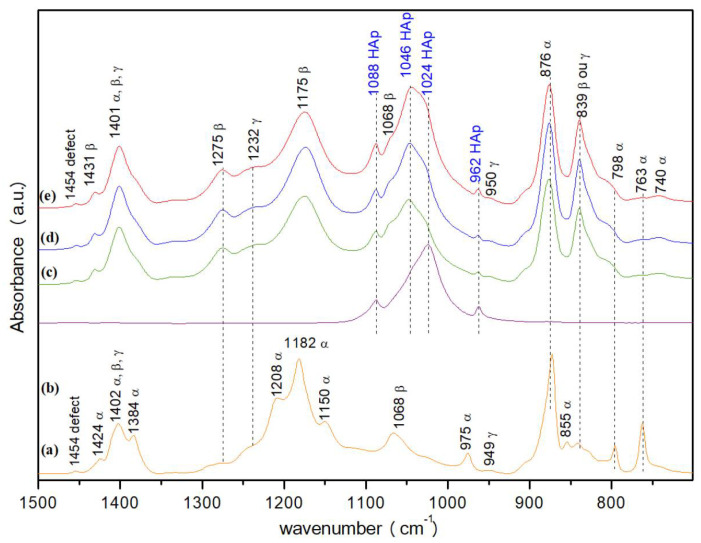
Absorbance ATR-FTIR spectra for (**a**) PVDF and (**b**) HA powders, (**c**) P14-H10, (**d**) P14-H15, (**e**) P14-H20 scaffolds.

Figure 5 shows the ATR-FTIR spectra for PVDF and HA powders and for the electrospun P14-H10, P14-H15, P14-H20 samples. In PVDF powder spectrum (Figure 5a), there was a greater presence of bands related to the α phase (763, 796, 855, 873, 975, 1150, 1182, 1208 and 1384 cm${}^{-1}$) than those related to the β phase (840, 1068, 1275 and 1424 cm${}^{-1}$) or to the γ one (950 and 1234 cm${}^{-1}$) [47,48,49,50]. In the HA powder sample spectrum bands at 962, 1024 and 1088 cm${}^{-1}$, characteristics of vibrational modes of the PO${}_{4}$${}^{3-}$ functional group of hydroxyapatite are observed [49,51]. The spectra related to the electrospun samples showed similar behavior among themselves. All the three spectra showed absence of bands at 855, 975, 1150, 1182, 1208 and 1384 cm${}^{-1}$, referring to the α phase, as well as the accentuation of the band at 840 cm${}^{-1}$, relative to the β or γ bands, and the band at 1275 cm${}^{-1}$, referring to the β phase, when compared with the spectrum of the PVDF powder. It is worth to note the appearance of an intense band, around 1175 cm${}^{-1}$, characteristic of the β phase [35] in the electrospun samples, which was not present in the spectrum of the powered sample. However, the great intensity of this band may have been caused by the overlap with the bands at 1150 and 1182 cm${}^{-1}$, referring to the α phase. There was also a significant decrease in the intensity of the bands at 763 and 796 cm${}^{-1}$, referring to the α phase, when comparing the spectrum of powdered PVDF with the spectra of electrospun samples.

All spectra showed bands around 950 and 1234 cm${}^{-1}$, referring to the γ phase, at 1402 cm${}^{-1}$, a band that can be attributed to any of the three phases mentioned here (α, β or γ), and in 1454 cm${}^{-1}$, related to the existence of a defect in the polymer chain. As for the bands related to hydroxyapatite, it is interesting to note that all the three spectra, in addition to presenting peaks at 962 and 1088 cm${}^{-1}$, also showed the emergence of a new peak at 1046 cm${}^{-1}$, related to the mode of vibration of the PO${}_{4}$${}^{3-}$ functional group. From HA [49], the band at 1024 cm${}^{-1}$appeared only as a shoulder. This may have occurred due to the overlapping of bands that occurred in this region of the spectrum. Perhaps the band at 1068 cm${}^{-1}$ (β phase of PVDF) contributed to the increase of the intensity of the band at 1046 cm${}^{-1}$, which should already have been present in the spectrum of the powdered HA sample (Figure 5a), but which was invisible because of the peak intensity at 1024 cm${}^{-1}$. Finally, it is observed that, as the proportion of HA in the samples increases, the peaks related to ceramics at 1046 and 1088 cm${}^{-1}$ also increase, when compared with the peaks referring to PVDF, as expected.

Observing the results listed in Table 1, P14 sample was the one which presented a greater amount of β phase when compared to the others. This result makes sense, since this solution had the lowest viscosity among the samples. This result demonstrates that the electrospinning process favors the transformation α→β of PVDF, which is extremely important for practical applications. During the electrospinning process, solutions with lower viscosity undergo greater stretching during flight of the solution jet, resulting in greater phase transition α→β due to the greater orientation of the polymer crystallites [44]. The method for calculating the beta phase was also applied for composites. Although the HA bands are present in the composite, the method could be used because there is no overlap of HA and PVDF bands in the spectrum regions used for these calculations. All the three electrospun samples showed very close β-phase percentage, with the P14-H15 sample showing the highest value. Despite that, the calculated β-percentages were lower than those obtained previously for the P14 sample, possibly due to the increase of the solution viscosity and the decrease of the solvent evaporation rate during the process.

**Figure 6:**
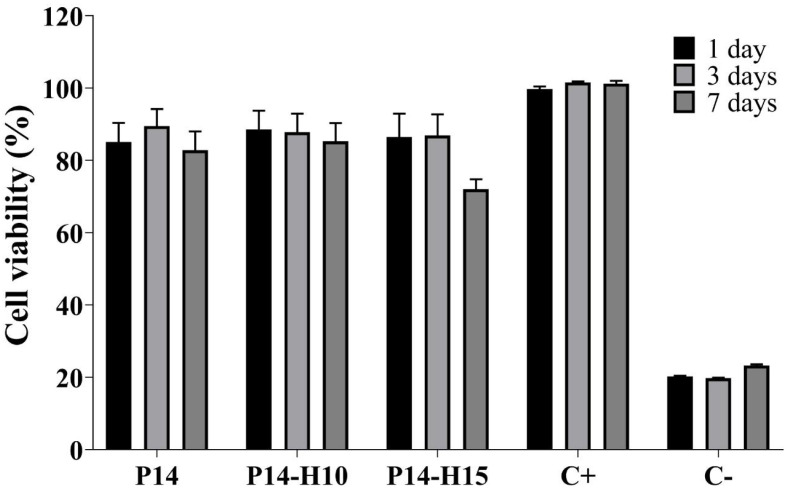
P14, P14-H10 and P14-H15 in vitro cytotoxicity assessment. (**a**) 1 day, (**b**) 3 days, and (**c**) 7 days. Neg: Negative control, fibroblasts cells without testing materials. Pos: Positive control, fibroblasts cells with 20% DMSO.

Figure 6 shows the results for the cytotoxicity analysis for periods of 1, 3 and 7 days of exposure of cells to electrospun membranes. The results show that the samples showed non toxicity and that the cells were able to grow and multiply on the surface of the membranes even after 7 days of incubation. For periods of 1 day and 3 days, the cell viability remained above 85% for all electrospun membranes. For the 7-days period, for P14 and P14-H10 samples the cell viability above 82% was observed, and only the P14-H15 sample showed cell viability of approximately 72%, which is still an excellent result, since this is a period considered long in cell viability tests. Thus, it was found that all analyzed samples showed low cytotoxicity even after 7 days of exposure by the direct method. This period is in fact, considered long in cell culture tests. These results show the great potential of this composite, in the form of electrospun membranes, to act in a biological environment. In fact, PVDF has been explored for the manufacture of composite biomaterials that are able of stimulating cell growth and differentiation. This is because the piezoelectric effect is able to stimulate certain cellular behaviors, such as proliferation and adhesion [52]. Braga et al. [53] had already demonstrated that PVDF/HA films performed adequately in indirect cytotoxicity tests performed in NCTC clone 929 cell culture medium, showing no cytotoxicity.

The 3D network of PVDF and HA composite scaffold fabricated in this present study exhibit adequate porosity, provides a good environment for the growth of fibroblasts and present a significantly higher percentage of the piezoelectric β-PVDF phase in relation to other studies involving PVDF and electrospinning [54,55]. The PVDF used in the present study has a significantly lower molecular weight than in these studies (approximately 4×). This difference may have also facilitated the transformation α→β of PVDF, representing a technological advantage of the P14-H10 and P14-H15 3D networks.

## 4. Conclusions

In this study, nano- and micrometric particles of hydroxyapatite (HA) were incorporated into PVDF solution (P14) in proportions of 10, 15 and 20% by mass (samples P14-H10, P14-H15 and P14-H20, respectively) processed by a simple single-fluid electrospinning. Scanning electron microscopy images revealed that there was an increase in fiber diameters when compared to those obtained for the pure polymer. P14 H10 and P14-H15 samples showed good results, with uniform fibers and good dispersion of HA particles. In order to characterize the crystalline phases of the polymer and HA, ATR analyzes were carried out on all samples. The results showed that there was a good incorporation of the ceramic in the electrospun membranes, with an increase in the characteristic bands of HA as the concentration of inorganic phase increased in the membranes. The percentage of β-PVDF phase reached 83.7 and 86.2% for P14-H10 and P14-H15 samples, respectively. P14, P14-H10 and P14-H15 samples were subjected to in vitro bioactivity tests in simulated body fluid (SBF) for periods of 7 to 21 days. After 21 days of immersion, the samples showed apatite growth, proving its bioactive character, being a promising candidate to act in the regeneration of bone tissue. These samples were also tested for their cytotoxicity. These tests were performed in human fibroblast cell culture medium by indirect and direct methods. The first had a maximum duration of 3 days, and the cells showed a survival rate very close to those obtained for the negative reinforcement. In the second, the maximum duration time was 7 days, and even after this period, which is considered a long period in cell culture tests, the membranes did not show any cytotoxicity characteristics. This result is very promising and indicates that these membranes may be used in the future as smart dressings for the regeneration of epithelial tissue.

## Figures and Tables

**Table 1:** β-phase amounts calculated for different PVDF-HA samples.

Sample	Percentage of HA	Percentage of β Phase
	(%)	(%)
PVDF (Powder)	0	32.6
P14	0	90.0
P14-H10	10	83.7
P14-H15	15	86.2
